# In Vitro Assessment on Designing Novel Antibiofilms of *Pseudomonas aeruginosa* Using a Computational Approach

**DOI:** 10.3390/molecules27248935

**Published:** 2022-12-15

**Authors:** Dian Rachmawati, Mochammad Zakki Fahmi, Muhammad Ikhlas Abdjan, Eddy Bagus Wasito, Imam Siswanto, Nurzafirah Mazlan, Jazirotur Rohmah, Afaf Baktir

**Affiliations:** 1 Faculty of Medicine, Universitas Airlangga, Surabaya 60132, Indonesia; 2 Supramodification Nano-Micro Engineering (SPANENG) Research Group, Universitas Airlangga, Surabaya 60115, Indonesia; 3 Department of Chemistry, Faculty of Science and Technology, Universitas Airlangga, Surabaya 60115, Indonesia; 4 Borneo Marine Research Institute, Universiti Malaysia Sabah, Kota Kinabalu 88400, Malaysia

**Keywords:** antibiofilm, *P. aeruginosa*, PMM/PGM, In Vitro, computational approach

## Abstract

An anti-biofilm that can inhibit the matrix of biofilm formation is necessary to prevent recurrent and chronic *Pseudomonas aeruginosa* infection. This study aimed to design compounds with a new mechanism through competitive inhibitory activity against phosphomannomutase/phosphoglucomutase (PMM/PGM), using in vitro assessment and a computational (in silico) approach. The active site of PMM/PGM was assessed through molecular redocking using L-tartaric acid as the native ligand and other small molecules, such as glucaric acid, D-sorbitol, and ascorbic acid. The docking program set the small molecules to the active site, showing a stable complex formation. Analysis of structural similarity, bioavailability, absorption, distribution, metabolism, excretion, and toxicity properties proved the potential application of ligands as an anti-biofilm. In vitro assessment with crystal violet showed that the ligands could reach up to 95.87% inhibition at different concentrations. The nitrocellulose membrane and scanning electron microscopic visualization showed that the untreated *P. aeruginosa* biofilm was denser than the ligand-treated biofilm.

## 1. Introduction

*Pseudomonas aeruginosa* (*P. aeruginosa*) is an environmental, opportunistic bacterium associated with various diseases with various clinical spectrums. It primarily causes ventilator-associated pneumonia (VAP) with high mortality/morbidity rates [1,2,3,4]. VAP-associated biofilm is resistant to antibiotics, and it has a protective capability from host immunity, thereby inducing recurrent and chronic infection. Thus, it is difficult to treat [5,6]. Novel strategies have been developed to manage *P. aeruginosa* biofilms, particularly in *Pseudomonas* biofilm-associated pneumonia.

Biofilm is a virulence factor of *P. aeruginosa*, which promotes bacterial resistance, thereby making bacteria more difficult to eradicate. Biofilm is a community of microorganisms encased in a matrix of extracellular polymeric substances (EPSs). Exopolysaccharides are essential components of EPS. Bacterial biofilms are sessile cellular communities. Hence, their properties differ from their free form (planktonic). Moreover, they are highly resistant to immune response and thousand times more resistant to antibiotics [6,7,8]. Antibiotics can kill planktonic bacteria; however, sessile bacteria in the form of biofilm must be eradicated with an anti-biofilm to ensure the life cycle of biofilm formation, the physiological properties of the bacteria, and the accessibility of biofilm. Furthermore, at present, no specific drugs have been developed for biofilm eradication.

*P. aeruginosa* biofilm contains three main exopolysaccharides: alginate, polysaccharide synthesis locus, and pellicle formation. They play an essential role in maintaining the structure and resistance of biofilm to antibiotics [8,9]. In mucoid *P. aeruginosa*, alginate plays a major role in immune response. Meanwhile, polysaccharide synthesis locus and pellicle formation are often found in the isolates of samples collected from people with infections caused by medical devices, such as catheters, thereby contributing to antibiotic resistance [8,10,11]. The synthesis of these exopolysaccharides is regulated via a pathway in which phosphomannomutase/phosphoglucomutase (PMM/PGM), whose activity is encoded by the algC gene, plays an important role [12]. In addition, PMM/PGM is involved in the lipopolysaccharide (LPS) biosynthetic pathway, which is another virulence factor of *P. aeruginosa* [13,14,15]. The homologs of several enzymes in the alginate biosynthetic pathway of *P. aeruginosa*, except for PMM/PGM, have been identified. The PMM/PGM enzyme plays an essential role in synthesizing exopolysaccharides as a biofilm extracellular matrix; therefore, developing the inhibitors of biofilm formation is a promising target [16].

PMM/PGM enzymes have a specific activity against two substrates, that is, mannose and glucose [13]. The PMM/PGM structure comprises four domains with one active site (the analog substrate) that can be a target of enzyme inhibitor drugs. The structural assessment of the PMM/PGM–substrate complex via X-ray crystallography was unsuccessful. Moreover, the PMM/PGM mutant structure that binds to the tartaric acid molecule on the active site (S108A) has been identified [16]. L-tartaric acid (TLA) accidentally binds to the active site of PMM/PGM when this compound is used as a precipitant in PMM/PGM crystallization. Hence, the structure of TLA is match to the active site of PMM/PGM, such that form a stable complex. Thus, TLA can be used as a template for exploring other ligands that potentially act as PMM/PGM competitive inhibitors.

The PMM/PGM is responsible in the synthesis of all exopolysaccharide components, namely Psl, Pel, alginate and lipopolysaccharide, for synthesis the extracellular matrix of biofilm. So by choosing PMM/PGM as the receptor target, it can be expected that the ligands that match this receptor in molecular docking study have strong anti-biofilm activity in the in vitro assessment. PMM/PGM inhibition will interfere with exopolysaccharide and LPS biosynthesis, thereby interrupting the permeability and stability of the protective barrier of *P. aeruginosa* and facilitating its elimination by the immune system and antibiotic penetration. This mechanism can be the target of designing an anti-biofilm. Meanwhile, anti-biofilm studies that have been carried target several mechanisms involved in biofilm formation, but no published studies have targeted PMM/PGM. Miari et al., (2021) revealed that natural products, that is, polysorbates, ginger, and wild blueberry, could decrease gene expression of exopolysaccharides and quorum sensing that encodes genes of *P. aeruginosa* [17].

Anti-biofilm strategies have been developed through several approaches such as anti-adhesive, aggressive antibiotic treatment, high-dose antibiotic combinations, and surgical removal. Martin et al. mentioned in their review that anti-biofilm agents have two targets, namely, bacterial cells and complex matrix [18]. However, investigation on the inhibition of the cellular matrix has not been intensively explored. In this study, although PMM/PGM was described as part of an enzyme encoded by a specific gene that could decrease gene expression, the target of anti-biofilm action did not directly lead to the inhibition of the enzyme.

Therefore, the development of anti-biofilm drugs, which are PMM/PGM competitive inhibitors based on substrate analogs, can increase the efficacy of the host immune system and antibiotic therapy, which are the primary modalities in treating *P. aeruginosa* biofilm-associated pneumonia. These results are essential in the further application of anti-biofilm agents after optimization. Accordingly, the current study aimed to assess the activity of PMM/PGM inhibitors against biofilm formation, which can serve as a basis for the future application of novel therapeutic strategies (combined antibiotics and competitive inhibitor-based anti-biofilm) against *P. aeruginosa*-associated pneumonia.

## 2. Results

### 2.1. Determination of the PMM/PGM Active Site via Redocking Analysis

**Figure 1:**
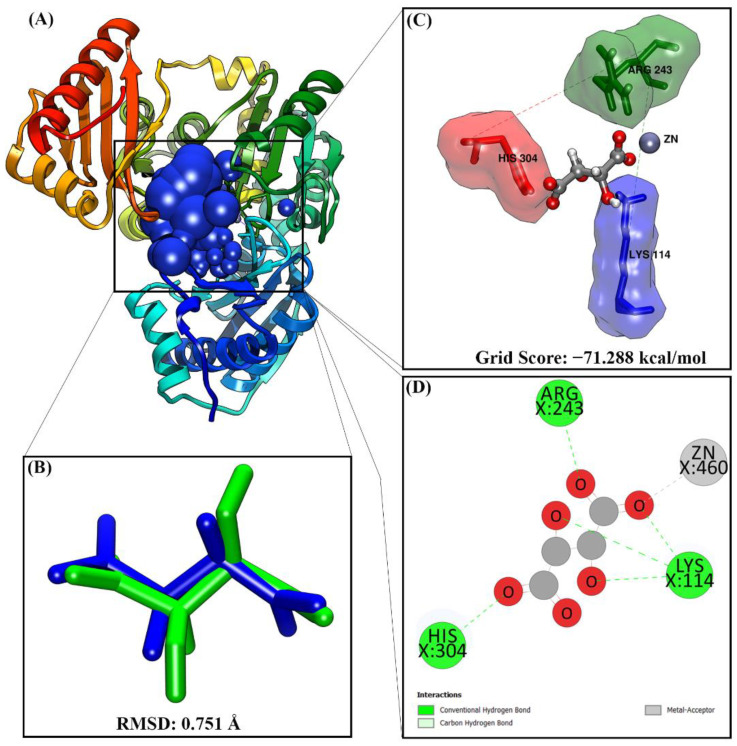
Determination of the receptor’s active site: (**A**) selection of a cluster sphere with a radius of 10.0 Å using the TLA coordinates; (**B**) the superposition of native ligands between the crystal (blue) and pose (green); (**C**) interaction between ligands (pose) and amino acid residues on the receptor’s active site; (**D**) type of interaction shown in the two-dimensional diagram.

The selected sphere cluster was taken at a radius of 10.0 Å from the TLA coordinates, and the cluster sphere could represent the active site receptor region (Figure 1A). Redocking analysis showed good superposition with the root mean square deviation (RMSD) value less than 2.0 Å (Figure 1B). In addition, the TLA coordinates obtained from redocking (pose) were similar to the TLA in the crystal form. Therefore, the RMSD value close to 0 Å indicated better pose coordinates.

TLA conformation showed an excellent binding affinity to a grid score of −71.288 kcal/mol (Figure 1C). In addition, ligand–residue interaction analysis at the receptor active site identified three amino acid residues (Lys114, Arg243, and His304) and one non-standard residue (Zn^+2^) as a cofactor of PMM/PGM (Figure 1C). Moreover, as presented in Figure 1D, ligand–receptor interactions had the following five hydrogen bonds: (1) O_3_…(Lys114)N-HZ2 at 2.48 Å, (2) O_1_…(Lys114)N-HZ_2_ at 2.29 Å, (3) O_2_…(Lys114)N-HZ_2_ at 2.14 Å, (4) O_4_…(His304)N-HE_2_ at 1.91 Å, and (5) O_11_…(Arg243)N-HHZ_1_ at 1.88 Å.

**Figure 2:**
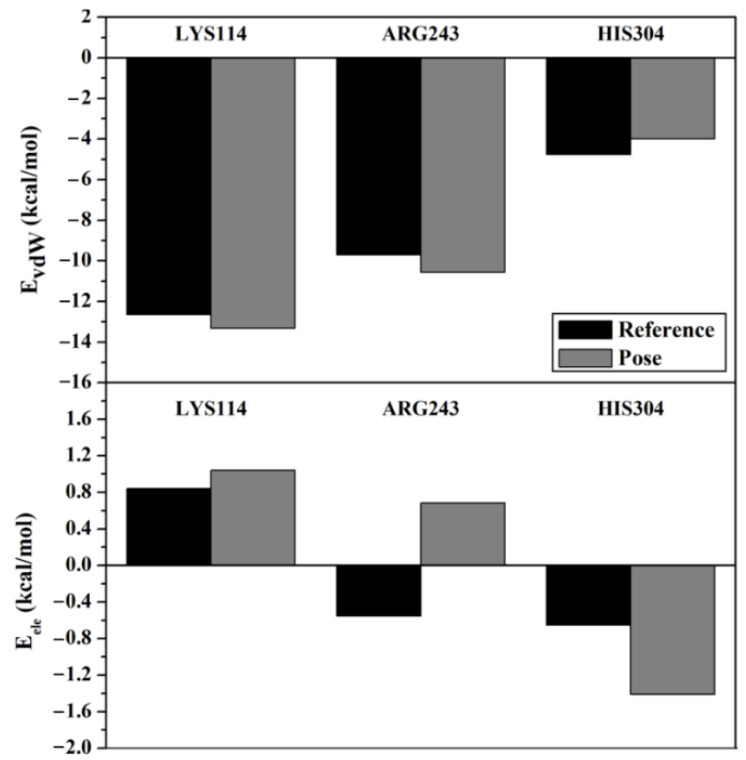
Footprint analysis of the primary amino acid residues responsible for the interaction between L-tartaric acid and phosphomannomutase/phosphoglucomutase.

E_vdW_ and E_ele_ indicated that electrostatic energy had the most significant contribution to binding affinity, and these two energy components in the native receptor–ligand interaction could be assessed via footprint analysis [17,19]. The analysis result was obtained by comparing each energy between crystal (reference) and redocked (pose) ligands (Figure 2).

Footprint analysis of three amino acid residues proved an excellent binding affinity, which is supported by the E_vdW_ + E_ele_ value (kcal/mol) for each residue, which was less than zero (negative value). For example, E_vdW_ and E_ele_ of TLA are −13.776 and −57.512 kcal/mol, respectively.

### 2.2. Ligand Exploration by Using TLA as Template

**Figure 3:**
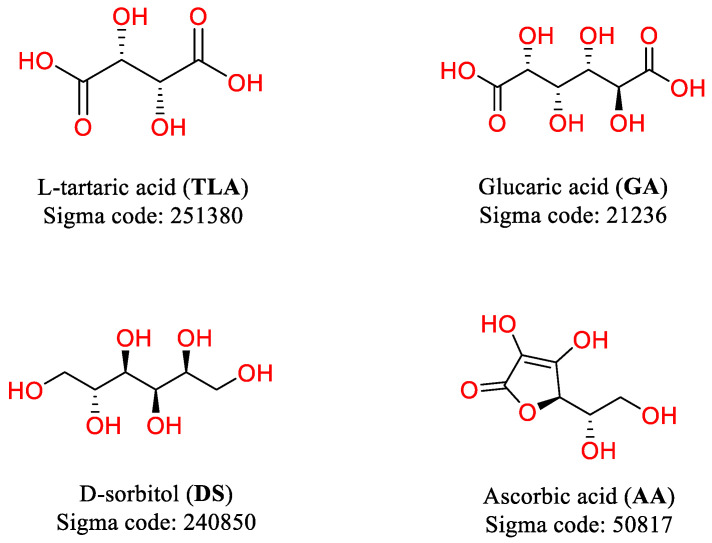
Phosphomanomutase/phosphoglucomutase competitive inhibitors as potential ligand candidates.

Based on redocking analysis of the active site of PMM/PGM and the grid score of TLA, we identified three ligands i.e glucaric acid [GA], D-sorbitol [DS], and ascorbic acid [AA] via ligand exploration based on the established criteria. These criteria include nontoxic, easy to obtain and reported safety for use (Figure 3).

### 2.3. Inhibitory Mechanism of TLA, GA, DS, and AA via Molecular Docking Analysis

**Figure 4:**
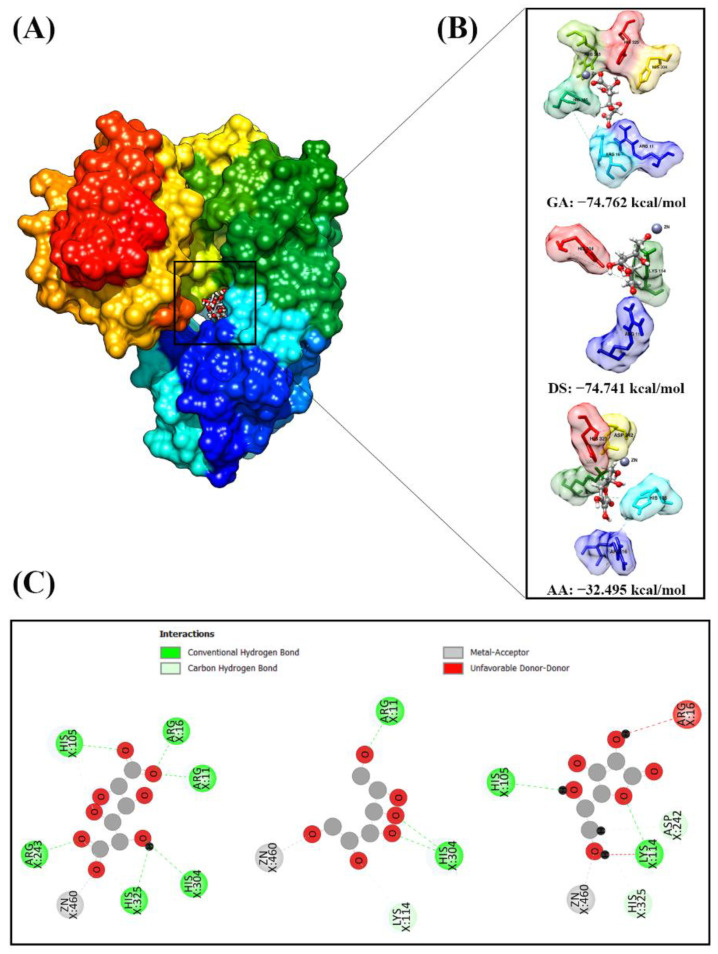
Phosphomanomutase/phosphoglucomutase (PMM/PGM) molecular docking: (**A**) docking of ligands on the PMM/PGM active site based on the L-tartaric acid (TLA) ligand coordinates as a reference, (**B**) conformation of ligands on the active site of PMM/PGM using the grid score function, and (**C**) the type of interaction shown in a two-dimensional diagram.

The grid score of each ligand was determined (TLA, −71.288; GA, −74.762; DS, −74.741; and AA, −32.495). This score is associated with the binding affinity (kcal/mol). The higher the negative grid score, the more vital the ligand interaction on the receptor’s active site from a thermodynamic aspect. The ligands and receptor showed conformation fitness, interacting via several receptor residues on the receptor’s active site, including GA-PMM/PGM (Arg11, Arg16, His105, Arg243, His304, His325, and Zn), DS-PMM/PGM (Arg11, Lys114, His304, and Zn), and AA-PMM/PGM (Arg16, His105, Lys114, Asp242, His325, and Zn). In particular, the hydrogen bond interaction with each ligand plays an important role in the target protein inhibition mechanism because the H-bond parameter is a strong category in ligand–receptor interactions [20,21]. The molecular docking analysis results showed potential H-bond interactions with amino acid residues to the receptor’s active site (Figure 4C), including GA-PMM/PGM GA-PMM/PGM ([1] O_5_…(Arg11)N-HH_2_ at 1.68 Å, [2] O_5_…(Arg11)N-HH_12_ at 2.26 Å, [3] O_5_…(Arg16)N-HH_12_ at 1.71 Å, [4] O_6_…(His105)N-HE_2_ at 3.05 Å, [5] O_7_…(Arg243)N-HE at 1.80 Å, [6] O_4_…(His304)N-HE_2_ at 2.90 Å, and [7] O_4_-H…(His325)NE_2_ at 2.07 Å), DS-PMM/PGM ([1] O_6_…(Arg11)N-HH_22_ at 1.74 Å, [2] O_3_…(His304)N-HE_2_ at 2.07 Å, and [3] O_4_…(His304)N-HE_2_ at 2.75 Å), and AA-PMM/PGM ([1] H_16_(O_5_)…(His105)ND_1_ at 2.59 Å and [2] O_8_…(Lys114)N-HZ_2_ at 1.99 Å).

**Figure 5:**
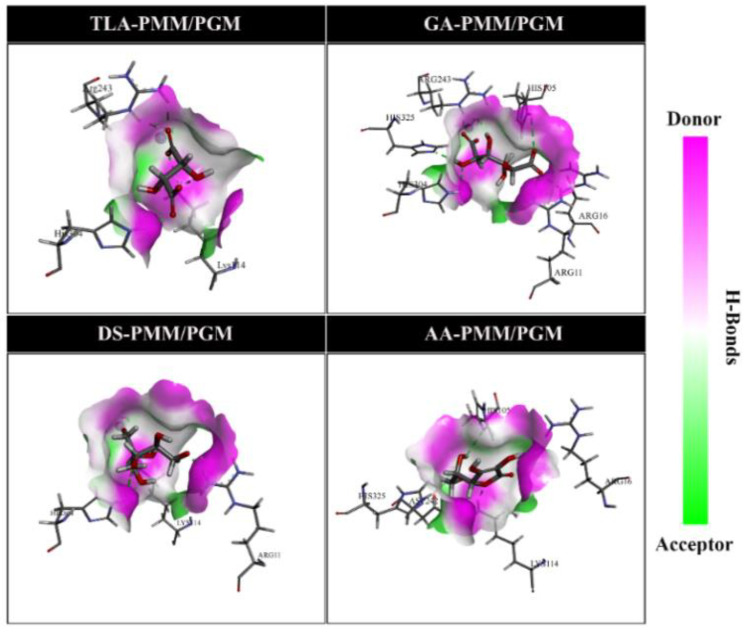
Hydrogen bonding pocket areas for molecular docking of phosphomannomutase/phosphoglucomutase by several ligands. Magenta and green indicate the hydrogen bond donor and acceptor on the pocket areas.

**Figure 6:**
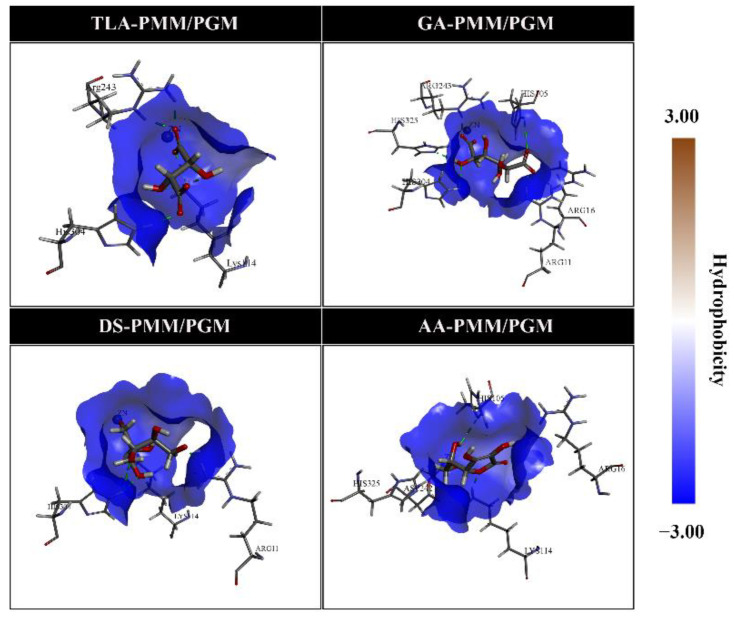
Hydrophobic pocket areas for molecular docking of phosphomannomutase/phosphoglucomutase by several ligands. Brown and blue indicate the hydrophobic and hydrophilic pocket areas, respectively.

H-bond interactions were further visualized in the form of a hydrogen bond acceptor (HBA) and hydrogen bond donor (HBD). As shown in Figure 5, the visualization was supported by classifying HBA and HBD areas on the receptor’s active site. Meanwhile, analysis of the hydrophobic area on the active site showed that each inhibitor was in a hydrophilic area (Figure 6). In addition, this result was indicated by a low hydrophobicity value (<0, blue area) for each ligand [22].

### 2.4. Prediction of Drug-Likeness, Bioavailability, Absorption, Distribution, Metabolism, Excretion, and Toxicity (ADMET)

Data on the prediction of drug-likeness of each ligand molecule with a competitive inhibitory activity against PMM/PGM based on docking analysis are shown in Table 1. All assayed molecules had good permeability as a potential anti-biofilm. However, one violation occurred in the topological polar surface area (TPSA) and HBD parameters for GA and DS inhibitors, respectively. Furthermore, pharmacokinetic analysis predicted that each ligand molecule had good permeability as a potential anti-biofilm because of the presence of only one violation. The violations occurred in TPSA and HBD for GA and DS, respectively.

**Figure 7:**
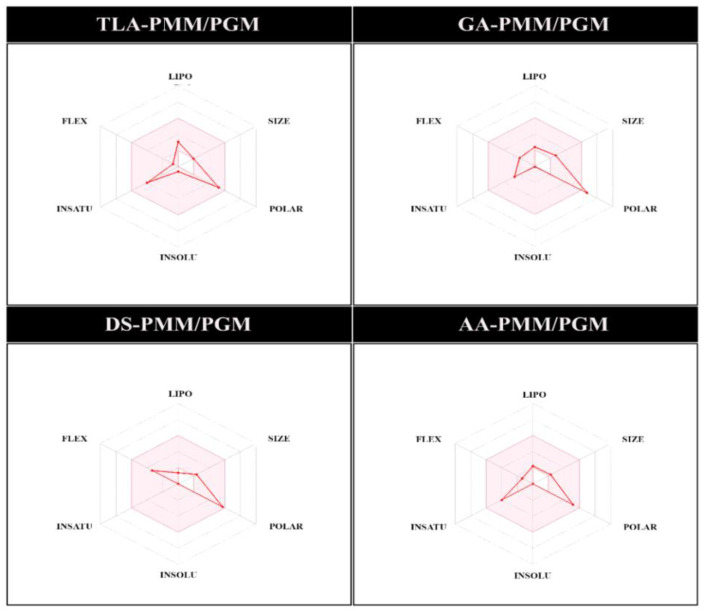
Prediction of the oral bioavailability of the phosphomannomutase/phosphoglucomutase inhibitor.

Figure 7 shows the prediction of oral bioavailability, which can provide theoretical information about the physicochemical properties of each potential anti-biofilm. The parameters measured included lipophilicity (−0.7 < X logP3 < 5.0), size (150 D < Mw < 500 D), polarity (20 Å^2^ < TPSA < 130 Å^2^), insolubility (0 < ESOL < 6), saturation (0.25 < Csp3 < 1), and flexibility (0 < rot bonds < 9) [23,24]. Furthermore, based on the prediction results, GA had one violation compared with TLA, DS, and AA (had no violation; Table 1 and Table 2). Overall, the bioavailability score shows that each inhibitor has good pharmacokinetic properties with a value of 0.55–0.56.

Table 3 shows the results of predictive analysis of ADMET. The prediction of absorption indicated that the anti-biofilm candidate had a low absorption ability on average, given its poor Caco-2 permeability and negative intestinal absorption—Human (HIA). However, AA had a positive HIA category > 30%, which indicated that AA is absorbed in the small intestine of humans. Distribution parameter analysis of each anti-biofilm candidate could predict blood–brain barrier (BBB) permeability (category: negative BB) with a log BB of less than −1.0. Therefore, all anti-biofilm candidates were not distributed, and they did not affect the central nervous system.

Meanwhile, the predicting metabolic parameters showed that the antibiofilm candidates did not inhibit cytochrome isoenzymes (CYP), including CYP1A2, CYP2C19, CYP2C9, CYP2D6, and CYP3A4. The absence of these isoenzymes indicated that each antibiofilm candidate was a promising drug candidate because it did not interfere with the CYP activity. Therefore, the antibiofilm candidate will not induce unwanted side effects on metabolic processes. In addition, the prediction of excretion showed that antibiofilm candidates were included in renal organic cation transporter-2 (OCT2) prediction, a subcategory of the non-substrate.

Analysis of toxicity showed remarkable results, where all antibiofilm candidates showed non-toxicity and met the following criteria: non-AMES toxic, non-hepatotoxicity, and non-skin sensitization [25]. Thus, overall prediction with the pkCSM server showed that all ligands had promising ADMET, which could be considered as drug candidates.

### 2.5. In Vitro Assessment of Ligands against P. aeruginosa Biofilm

#### 2.5.1. Microtiter Plate Assay

The activity of each ligand as an anti-biofilm was evaluated at several concentration ranges using crystal violet assay. Based on the data presented in Table 4, one of the ligands could reach up to 95.87% inhibition at different concentrations. The minimal biofilm inhibitory concentration (MBIC) of TLA had >50% anti-biofilm activity at 2 × 10^3^ µg/mL. By contrast, GA or calcium D-saccharate tetrahydrate had >50% inhibitory biofilm formation activity at 2.5 × 10^2^ µg/mL.

**Figure 8:**
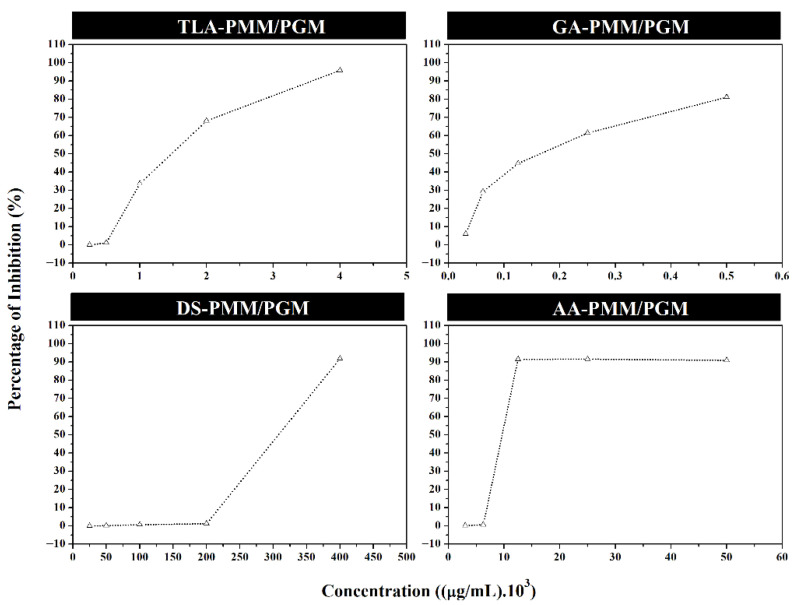
Inhibitory activity of *Pseudomonas aeruginosa* biofilm formation by TLA, glucaric acid (GA), D-sorbitol (DS), and ascorbic acid (AA) in vitro using the crystal violet method on 96-well microtiter plates.

DS had a >50% inhibitory effect on biofilm formation at 4 × 10^5^ µg/mL. AA had a >50% inhibitory activity against *P. aeruginosa* biofilm formation at 1.25 × 10^4^ µg/mL, and its inhibitory activity reached 91.5% (Figure 8).

#### 2.5.2. Antibiofilm Assessment of *P. aeruginosa* on the Nitrocellulose Membrane

**Figure 9:**
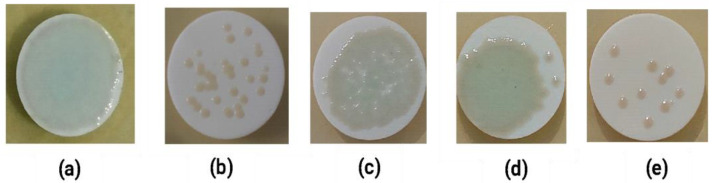
Macroscopic appearance of *Pseudomonas aeruginosa* biofilm treated with L-tartaric acid (TLA), glucaric acid (GA), D-sorbitol (DS), and ascorbic acid (AA). (**a**) Blank (control), (**b**) TLA, (**c**) GA, (**d**) DS, and (**e**) AA.

Figure 9 shows the macroscopic morphology of the *P. aeruginosa* biofilm treated with each ligand upon the nitrocellulose membrane. Moreover, each ligand-treated biofilm was smaller and thinner than the biofilm produced in the control experiments without ligand treatment.

#### 2.5.3. Scanning Electron Microscopic Visualization of Ligand-Treated *P. aeruginosa* Biofilm

**Figure 10:**
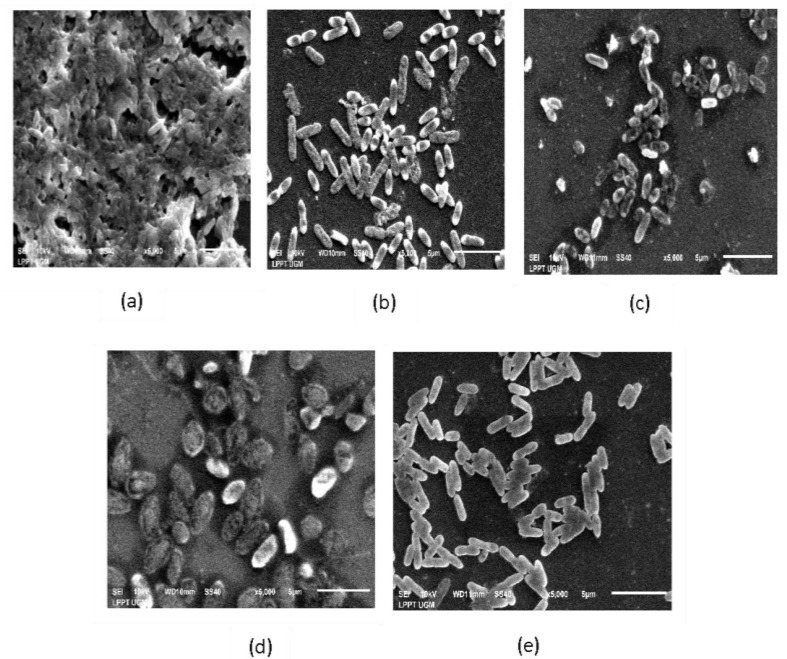
*Pseudomonas aeruginosa* biofilm on glass coverslips was monitored using a scanning electron microscope with a magnification of ×5000: (**a**) control (untreated) cells, (**b**) treated with L-tartaric acid (TLA), (**c**) glucaric acid (GA), (**d**) D-sorbitol (DS), and (**e**) ascorbic acid (AA).

Figure 10 shows the appearance of *P. aeruginosa* biofilm under an electron microscope. All ligand-treated biofilms are different from the control (untreated).

Figure 10a shows that the untreated *P. aeruginosa* biofilm comprised bacterial cells with some exopolysaccharides (EPSs). The bacterial cell walls appeared intact and closed together, attached, and covered by an EPS matrix. Figure 10b–e shows the ability of ligands to reduce biofilm formation. The bacterial cells were grown on coverslips for 8 h in the attachment stage, continually treated with the addition of ligands in Brain Heart Infusion Broth (BHIB) media, and incubated for 24 h.

## 3. Discussion

Rodrigues et al., (2017) showed the characteristics of polymicrobial biofilms associated with pneumonia, with bacterial–fungal polymicrobial biofilms colonizing the endotracheal surface. *P. aeruginosa*, which is commonly associated with pneumonia, interacts and interplays with other microbes in polymicrobial biofilms, thereby enhancing pathogenesis and affecting antimicrobial therapy. Therefore, the high mortality/morbidity rate associated with pneumonia and the increased antibiotic resistance worldwide have prompted studies on novel therapeutic strategies to combat polymicrobial infection in pneumonia. Rodrigues et al., (2017) proposed combinational antimicrobial therapy using one antibiotic (polymyxin B, PolyB) and anti-fungal (amphotericin B, AmB) agent against the polymicrobial biofilms of *P. aeruginosa* and *Candida albicans* [2]. Meanwhile, we developed an antimicrobial therapy with an anti-biofilm to eradicate the polymicrobial biofilms of *P. aeruginosa* and *C. albicans*. In addition, we evaluated the performance of several anti-biofilms of *C. albicans* that enhance the anti-fungal action of fluconazole [26].

The exploration of several *P. aeruginosa* anti-biofilms based on the competitive inhibitors of the PMM/PGM enzyme, which is responsible for extracellular matrix biosynthesis, was assessed. In the present study, substances with a structure similar to that of the PMM/PGM active site were identified, which served as ligands for this enzyme and a macromolecule. The ligands included the stereoisomer of the native substrate of PMM/PGM, mannose, and glucose and the ligand of S108A PMM/PGM (TLA). The results showed that three substances (AA, GA, and DS) matched the criteria. Based on the structure similarity between these ligands and TLA (as the natural substrate of PMM/PGM), the ligands should occupy the active site of PMM/PGM. Thus, these ligands may induce activity as PMM/PGM competitive inhibitors, which can be predicted via in silico analysis, thereby indicating its activity through in vitro assay.

Theoretical studies on the use of substrate analogs as PMM/PGM inhibitors based on their structural similarity (mimic) to native ligands and their commercial availability can provide an efficient approach to drug design. In addition, the mechanism of PMM/PGM competitive inhibition was analyzed on the basis of the interaction of the inhibitor with residues on the receptor’s active site at a molecular level. Redocking determined the active site of PMM/PGM based on its interaction with TLA as the native ligand. Moreover, sphere cluster selection using Dock6 can facilitate easier identification of native ligand coordinates at the receptor active site [27,28].

Docking of the PMM/PGM ligand was conducted at the initial coordinates of the redocking results (Figure 4A). In addition, ligands with a binding affinity of <0 (kcal/mol) have good interactions with receptors or targeted proteins [19,20,27]. The higher the negative value, the more robust the interaction with the inhibitor. Redocking had good feasibility in determining the active site of the receptor; thus, it could be used for docking other ligands. Molecular docking analysis of the PMM/PGM ligands showed that each ligand had an excellent conformation with a negative grid score.

The performance and characteristic prediction of competitive inhibitors were investigated via in silico pharmacokinetic studies. Furthermore, the pharmacokinetics of inhibitors were evaluated via several parameters, including drug-likeness, bioavailability, and ADMET. These parameters can provide information on biological activities based on their structure. For example, drug-likeness analysis observes drug permeability by considering Lipinski’s and Veber’s rules. A drug candidate has good permeability if it does not deviate by more than one Lipinski’s and Veber’s rule [29].

Previous pharmacokinetic studies confirmed that each inhibitor meets the criteria of oral drugs, showing that the development of an excellent oral drug had ≤2 deviations [29]. Therefore, this information will be considered in selecting potential inhibitors based on oral bioavailability. Consequently, in silico pharmacokinetics study resulted in potential antibiofilms of *P. aeruginosa*. Then, this prediction has been proven via quantitative and qualitative in vitro assays.

The first in vitro assay using the crystal violet method confirmed that the four ligand compounds could inhibit *P. aeruginosa* biofilms. Using spectrophotometric analysis and the crystal violet method, biofilm formation was determined on the basis of optical density and [30,31]. In vitro experiments showed the potential application of these compounds as anti-biofilm agents.

Tartaric acid derived from ascorbic acid as the native ligand represents aldaric acid, which can be found in different plants. Tartaric acid and its salts (K, Na, Na/K, and calcium tartrate) are widely used in the food industry as flavoring agents, food additives, emulsifiers, antioxidant synergists, and sequestrates, which are safe for humans [32,33]. Crystal violet assay shows that TLA at 4 × 10^3^ µg/mL inhibits biofilm formation by almost 100%. This result indicates the application potential of TLA as an anti-biofilm candidate against *P. aeruginosa* biofilm. Previous studies reveal that tartaric acid and its derivates have antimicrobial activity against several bacteria [34,35], but no published data have shown that tartaric acid can be an anti-biofilm candidate.

Walaszek (1997) reported that GA has anticancer and antioxidant properties. However, its anti-biofilm and antibacterial activities have not been evaluated. Calcium D-glucarate is a supplement for cancer prevention, liver detoxification, and hormone regulation. Moreover, it is a calcium salt of D-GA, a non-toxic compound found in several fruits and vegetables, particularly grapefruit, apples, oranges, and broccoli [36,37]. The in vitro anti-biofilm test of GA was constrained by the presence of calcium compounded with the active ingredient (GA). Hence, its solubility in the aquadest solvent was low. However, calcium-bound GA could be assayed at a maximum concentration of 5 × 10^2^ µg/mL. GA in this assay has shown an inhibitory activity of >50% when the concentration is below 5 × 10^2^ µg/mL.

Sorbitol, a polyol group, inhibited biofilm formation in *S. mutant* bacteria [38]. The polyol group has an osmotic effect that causes osmolarity to remain constant at each concentration tested without any significant effect on absorbance. Abbas et al., (2012) showed that sorbitol could inhibit *P. aeruginosa* biofilm formation at 4 × 10^5^ µg/mL. Sorbitol is sugar alcohol with a sweet taste which is slowly digested in humans. This compound can be produced by reducing glucose, which converts the aldehyde group (-CHO) into primary alcohol or the hydroxyl group (-C(OH)H_2_). Sorbitol is widely found in fruits, and it is used in industries as an additive to food, cosmetics, and medicine [39].

The inhibitory ability of AA describes the mechanism of PMM/PGM inhibition via substrate analogs. Several studies have shown the capability of AA to interfere with the physiology of microorganisms. For example, Novak and Fratamico (2004) reported the capability of AA to inhibit quorum sensing in *C. perfringens.* Meanwhile, Serry et al., (2008) showed that AA served as an efflux pump inhibitor. Finally, Abbas et al., (2012) showed that sodium ascorbate inhibited planktonic growth in *P. aeruginosa* at 5–20 mg/mL.

The second in vitro assay used the colony biofilm method. This method is suitable for assessing the strength of an anti-biofilm or resistance to antibiotics. The nitrocellulose membrane is a good surface for growing *P. aeruginosa* biofilms [40]. Biofilm preparation using this method can promote the supply of new nutrients by simply transferring membrane-grown cells to fresh agar plates. Therefore, one can easily change the carbon source or the type of anti-biofilm treatment without washing the cells. Figure 9 shows that the macroscopic appearance of each ligand was different from that of the control. The untreated *P. aeruginosa* biofilm was denser than that of the ligand-treated biofilm. Based on this qualitative experiment, each test ligand reduced the formation of *P. aeruginosa* biofilms. More detailed evaluations of microscopic structures were conducted by scanning electron microscopy (SEM).

The third in vitro assay was performed using an electron microscope. Figure 10 shows that biofilm formation by *P. aeruginosa* on glass coverslips was monitored via SEM. This section showed that the cell morphology has changed with ligand treatment. The preparation of SEM comprises several steps that can prevent changes in the morphological structure, although the cell undergoes death [41].

SEM analysis revealed that untreated *P. aeruginosa* biofilms (control) comprise bacterial cells and an EPS matrix. *P. aeruginosa* biofilms grown in vitro are formed through several stages. First, planktonic bacteria attach to abiotic or biotic surfaces through flagella and adhesion. Second, an irreversible attachment will occur with the help of the SadB protein. Third, microcolonies are formed in the complex layer of biomolecules and the EPS matrix. Fourth, the biofilm matures. Fifth, bacteria are released from the biofilm to form a new biofilm [10].

All tested ligands were added after the first stage when bacterial cells were attached to the coverslips’ surface. Based on the stages of biofilm formation [10], the EPS matrix will begin to form in the second stage. Therefore, ligands were added to inhibit this process through their function as a substrate analog of PMM/PGM. The TLA with a concentration of 4 × 10^3^ µg/mL showed sparse cells without EPS, which is similar to the addition of GA (5 × 10^2^ µg/mL), DS (4 × 10^5^ µg/mL), and AA (5 × 10^4^ µg/mL). The appearance of the bacterial cell wall in each treatment varied. The TLA and GA-treated cells and the cell walls of some bacteria were not intact and lysed. Meanwhile, the DS-treated cells were affected by osmotic pressure, thereby causing the cells to become turgid. Meanwhile, the cell walls remained intact. However, EPS was not found in AA-treated cells.

## 4. Materials and Methods

### 4.1. Materials and Bacteria

The ligands used are commercially available, i.e., TA (L-Tartaric acid, Sigma-Aldrich, St. Louis, MO, USA), GA (Calcium D-saccharate tetrahydrate/D-Glucaric acid, Sigma-Aldrich, St. Louis, MO, USA), DS (D-sorbitol, Sigma Aldrich, St. Louis, MO, USA) and AA (L-Ascorbic acid, Sigma Aldrich, St. Louis, MO, USA). *P. aeruginosa* was isolated from the sputum of patients with pneumonia. It was identified using VITEK 2 COMPACT (Biomerieux, Marcy-I’Étoile, France) in Clinical Microbiology Laboratory DR. SOETOMO Hospital Surabaya (Lab. Number 10895) and was assayed to identify its biofilm formation capability. This isolate was stored at −80 °C in Tryptone Soya Broth (TSB) CMO129 (Oxoid, Basingstoke, UK) supplemented with glycerol 20% (Merck, Darmstadt, Germany). Before each assay, *P. aeruginosa* frozen stocks were freshly cultured to brain heart infusion agar and then incubated aerobically at 37 °C for 18–24 h.

### 4.2. Ligands as PMM/PGM Inhibitors

The PMM/PGM S108A mutant was used as the target protein to design the *P. aeruginosa* anti-biofilm, which was retrieved as a crystal structure (1K2Y) from the Protein Data Bank (PDB) (rscb.org/pdb) accessed on 23 August 2019. TLA was a PMM/PGM native ligand extracted from the 1K2Y crystal structure, which was used as a reference coordinate in determining the active site of PMM/PGM [16]. In addition, TLA, GA, DS, and AA had a structure similar to the substrate, and they were commercially available in the Sigma database. Ligand selection begins with exploring compounds in https://pubchem.ncbi.nlm.nih.gov/ (accessed on 4 September 2019) that have similar structure to TLA, includes information about related compounds, chemical vendors, drug and medication information, food additives and ingredients, pharmacology and biochemistry, use and manufacturing, safety and hazards and toxicity. Ligands structure similar to TLA are hypothesized to have the same, or better, activity. The inhibitory activity of selected ligands against PMM/PGM were then screened and evaluated through molecular docking analysis and considered for commercial availability, so that they can proceed to in vitro experiment.

### 4.3. Receptors and Ligands

PMM/PGM served as the receptor during docking. The receptor was prepared by extracting amino acid residues and Zn^+2^ atoms from the molecular structure of PMM/PGM using Chimera version 1.13. The ligand geometry was optimized using Semi-empirical Quantum Method-Parametric Method 3 (SQM-PM3). Further optimization was performed with HyperChem 8.0 to calculate the electrostatic potential charge of each atom. Analysis was continually performed to determine the ligand and receptor parameters such as bonded, non-bonded, and charged using the AMBER FF14SB force field tool [42] and Austin Model 1-Bond Charge Correction [43] contained in Chimera version 1.13.

### 4.4. Molecular Docking

Molecular docking was performed in two stages, namely, redocking and docking of other test ligands, using the Dock6 package. During redocking, a cluster sphere with a radius of 10.0 Å was first selected to determine the native TLA ligand coordinates on the receptor active site. Next, a grid box with a grid spacing of 0.1 Å was created on the selected cluster sphere around the receptor active site. The native ligand RMSD value represented successful redocking (≤2.0 Å) [44]. Next, binding affinity and footprint were determined using the grid score function with a rigid conformation. The scoring function could clearly describe the conformation (pose) between the ligand and the receptor [27]. In addition, the grid score function could predict several energy contributions, including van der Waals energy (E_vdW_) and electrostatic energy (E_ele_).

### 4.5. Pharmacokinetic Study

The pharmacokinetic properties of each ligand were predicted using the SwissADME (http://www.swissadme.ch/index.php) (accessed on 13 July 2020) and pkCSM (http://biosig.unimelb.edu.au/pkcsm/prediction) websites (accessed on 13 July 2020), with a 2D ligand structure (SMILES format) [23,45] Drug-likeness and bioavailability were also predicted using SwissADME by analyzing Lipinski’s and Veber’s rules. In addition, ADMET could be calculated via pkCSM analysis. The ligand properties could predict whether a ligand has a biological activity suitable as a viable drug candidate.

### 4.6. In Vitro Assay of Biofilm Matrix Extracellular Inhibition

#### 4.6.1. Microtiter Plate Assay

The capability of the ligand to inhibit the extracellular matrix formation of *P. aeruginosa* biofilm was assayed using the crystal violet method on a sterile flat bottom 96-well microtiter plate (Corning Costar 3596, Life Sciences, New York, NY, USA) and indicated as Minimal Biofilm Inhibitory Concentration (MBIC).

Each well was filled with 95 µL of Brain Heart Infusion (BHI) broth (Conda Pronadisa, Madrid, Spain) and 5 µL of *P. aeruginosa* inoculum 10^7^ Colony Forming Unit (CFU/mL). The microtiter plate was incubated for 8 h to achieve irreversible adherence. Then, the medium was discarded and replaced with the ligands. Each well was filled with 100 mL of medium, and each ligand was investigated at twofold increasing concentrations. The starting concentrations were determined on the basis of the preliminary test result of each ligand, there were TLA (1.25 × 10^2^–1.6 × 10^4^ µg/mL), GA (3 × 10^1^–2 × 10^3^ µg/mL), DS (3 × 10^3^–4 × 10^5^ µg/mL), and AA (3.9 × 10^2^–5 × 10^4^ µg/mL).

Next, the microtiter plate was incubated for 24 h at 37 °C. This assay used meropenem (Minimum Inhibitory Concentration/MIC: 0.5 µg/mL) as the positive control and bacterial inoculum as the negative control. The treatment was replicated three times.

After incubation, the microtiter plate was washed with distilled water three times and then dried at room temperature for 5 min. Next, 125 µL of 1% crystal violet solution was added to each well and then incubated at room temperature for 15 min. The microtiter plate was washed three times under running water. Next, 200 µL of ethanol was added to each well [30,46]. Optical density was measured at a wavelength of 595 nm. Then, the MBIC was calculated in accordance with the following formula:

$$\%\;\mathrm{inhibition}=\frac{{(\mathrm{OD}}_{\;\mathrm{growth}\;\mathrm{control}}-{\;\mathrm{OD}}_{\mathrm{sample}})\;}{\mathrm{OD}\;\mathrm{growth}\;\mathrm{control}}\;\times \;100$$

#### 4.6.2. Colony Biofilm Assay

*P. aeruginosa* was cultured on 13 mm diameter nitrocellulose semipermeable membrane (Whatman, Buckinghamshire, UK) and placed on an agar plate at 36 °C. The preparation of the ligand test was similar to the previous in vitro method. In total, 20 µL of bacterial suspension that reacted with the optimal concentration of each ligand was dripped onto a nitrocellulose semipermeable membrane layer on BHI agar and incubated for 24 h. The macroscopic morphology of the biofilm was observed on the membrane surface.

#### 4.6.3. Surface Morphological Investigation

*P. aeruginosa* biofilms were formed on a sterile 12 mm diameter round glass coverslip (Electron Microscopy Sciences, Hatfield, PA, USA) in a 24-well polystyrene microtiter plate. The preparation of biofilm formation and ligand treatment was similar to the previous procedures in the in vitro biofilm matrix extracellular inhibition assay. The coverslips were submerged in 400 µL of bacteria and ligand suspension and incubated overnight at 37 °C. A biofilm grown without ligand treatment was used as the control. SEM preparation was performed in accordance with the method of Hess et al. and Kazmierczak [41,47] with modifications. First, the suspensions were discarded and rinsed two times with 1% sterile Phosphate Buffered Saline (PBS) (G-Biosciences, St. Louis, MO, USA). Then, the 12 mm of coverslips were fixated with 200 µL of 2.5% (b/v) glutaraldehyde in 0.15 M cacodylate buffer (Electron Microscopy Sciences, Hatfield, PA, USA) overnight at room temperature. Furthermore, after the fixative agents were discarded, 200 µL of methanol (Merck, Branchburg, NJ, USA) was added for 30 min during dehydration. Finally, the coverslip was removed from wells, dried overnight, and coated with 99.9% Au using a JEOL JEC-3000FC auto fine coater for 120 s 3,4 Pascal. Then, a double-sided carbon tape was attached for examination via SEM (JEOL JSM-6510LA).

## 5. Conclusions

The in vitro experiments in this study proved that TLA, GA, DS, and AA had excellent activity against the biofilm of *P. aeruginosa*. These potential anti-biofilm agents can reach almost 100% inhibition values at different concentrations. TLA, GA, DS, and AA had a biofilm inhibition rate of >50% at 2 × 10^3^, 2.5 × 10^2^, 4 × 10^5^, and 1.25 × 10^4^ µg/mL, respectively. Nitrocellulose membrane assay showed that controls and ligand-exposed biofilm had different appearances. SEM images revealed that the control biofilm could be covered in the EPS matrix. However, these images were not visible in treated biofilms. Molecular docking analysis showed that each ligand had an excellent binding affinity to the receptor, as supported by several hydrogen bonds (i.e., TLA [5 H-bonds act as HBA], GA [6 H-bonds act as HBA and 1 H-bond act as HBD], DS [3 H-bonds act as HBA], and AA [1 H-bond act as HBA and 1 H-bond act as HBD]). Furthermore, each inhibitor can be a promising drug candidate based on its pharmacokinetic properties. Analysis of drug-likeness and bioavailability showed good results, where all anti-biofilm candidates showed non-toxicity and met the following criteria: non-AMES toxic, non-hepatotoxicity, and non-skin sensitization.

## Figures and Tables

**Table 1:** Drug-likeness analysis based on Lipinski’s rule and Veber’s rule.

Code	Lipinski’s Rule ^(a)^				Veber’s Rule ^(b)^		Validations/n
	M LogP	MW (g/mol)	∑ HBD	∑ HBA	∑ Rotatable Bonds	TPSA (Å^2^)	
TLA	−2.37	148.07	2	6	1	108.74	0
GA	−3.17	208.12	4	8	3	149.20	1
DS	−2.77	182.17	6	6	5	121.38	1
AA	−2.60	176.12	4	6	2	107.22	0

^(a)^ Lipinski’s rule criteria: M LogP ≤ 4.15, MW ≤ 500 Dalton, ∑ HBD ≤ 5, and ∑ HBA ≤ 10. ^(b)^ Veber’s rule criteria: ∑ rotatable bonds ≤ 10 and TPSA ≤ 140.00 Å^2^.

**Table 2:** Oral bioavailability properties of TLA, GA, DS, and AA.

Code	Log *P*_o/w_ (X LogP3)	Log S (ESOL)	Fraction Csp3	Bioavailability Score
TLA	−0.12	−0.62	0.50	0.55
GA	−1.35	−0.08	0.67	0.55
DS	−3.10	1.31	1.00	0.55
AA	−1.64	0.23	0.50	0.56

**Table 3:** Absorption, distribution, metabolism, excretion, and toxicity (ADMET) prediction properties using the pkCSM server.

Parameters	TLA	GA	DS	AA
Absorption				
Caco-2 permeability (log Papp in 10^−6^ cm/s)	−0.71	−1.10	−0.74	−0.30
Intestinal absorption-human (% absorbed)	0	0	15.68	55.13
Distribution				
BBB permeability (log BB)	−1.26	−1.81	−1.58	−1.13
Metabolism				
CYP1A2 inhibitor	No	No	No	No
CYP2C19 inhibitor	No	No	No	No
CYP2C9 inhibitor	No	No	No	No
CYP2D6 inhibitor	No	No	No	No
CYP3A4 inhibitor	No	No	No	No
Excretion				
Renal OCT2 substrate	No	No	No	No
Toxicity				
AMES toxicity	No	No	No	No
Hepatotoxicity	No	No	No	No
Skin sensitization	No	No	No	No

High-Caco-2 permeability: log Papp > 0.90 and poor Caco-2 permeability: log Papp < 0.90. Intestinal absorption-human (+): HIA > 30% and intestinal absorption-human (−): HIA < 30%. BBB permeability (+): log BB > 0.3 and BBB permeability (−): log BB less than −1.0.

**Table 4:** In vitro study of ligand activity as an extracellular matrix biofilm inhibitor of *Pseudomonas aeruginosa*.

L-Tartaric Acid		Glucaric Acid		D-Sorbitol		Ascorbic Acid	
Dose (µg/mL) × 10^3^	% Inhibition	Dose (µg/mL) × 10^3^	% Inhibition	Dose (µg/mL) × 10^3^	% Inhibition	Dose (µg/mL) × 10^3^	% Inhibition
0.25	0	0.03	6.1	25	0	3.125	18.45
0.5	1.14	0.06	29.3	50	0.2	6.25	63.76
1	33.64	0.125	44.75	100	0.7	12.5	91.5
2	68.11	0.25	61.43	200	1.25	25	91.6
4	95.87	0.5	81.18	400	91.87	50	90.9

## Data Availability

All data that support the results of this study are available from the corresponding author upon reasonable request.
